# Assessing Science Practices in Undergraduate Chemistry Laboratories: Why We Need to Do Better—and How We Should

**DOI:** 10.1021/acs.jchemed.5c00988

**Published:** 2025-12-30

**Authors:** Vinay Bapu Ramesh, Michael K. Seery, Renee Cole

**Affiliations:** † Department of Chemistry, 4083 University of Iowa, Iowa City, Iowa 52242, United States; ‡ Centre for Academic Language and Development, University of Bristol, Bristol, BS8 1QU, U.K.

**Keywords:** General Public, First-Year Undergraduate/General, Second-Year Undergraduate, Upper-Division Undergraduate, Curriculum, Laboratory Instruction, Testing/Assessment, Student-Centered Learning

## Abstract

An undergraduate chemistry laboratory should serve as a space where students learn how to *do* chemistry. Laboratory time should not be used primarily to reinforce content knowledge but rather to develop essential competencies, including scientific practices. To support the acquisition of these competencies, students must be provided with meaningful opportunities to engage with scientific practices, and assessments must explicitly evaluate these practices, given the critical role assessment plays in shaping student learning. This article outlines several challenges associated with fostering engagement with scientific practices in laboratory settings, reviews existing assessment tools and their limitations, and proposes approaches to address some of these issues. We argue that there is a need for flexible assessment approaches, applicable across a range of undergraduate chemistry laboratory contexts. Properly designed assessment tools can also serve as practical guides for instructors seeking to make the laboratory a space primarily dedicated to skill development.

## Graphical abstract

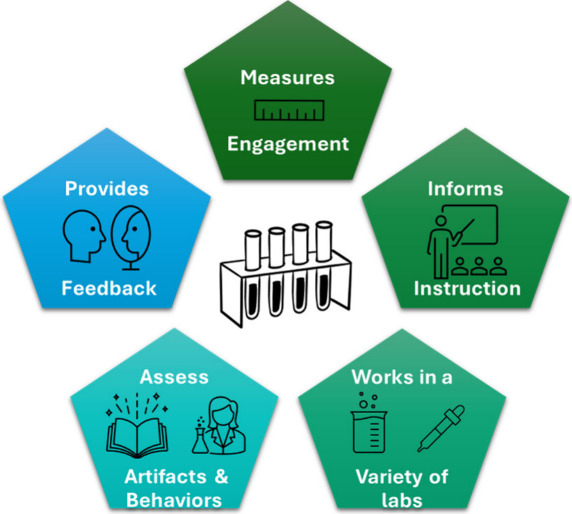

## Introduction

What it means for students to develop expertise in a discipline is a complex question to answer and often depends on the unique needs of that discipline. In chemistry, education researchers and instructors have pondered this question (and offered various solutions) for over a hundred years.^1^ In 2012, the National Research Council (NRC) put forth a framework for K-12 science education (*the NRC Framework*) that emphasizes three-dimensional learning: disciplinary core ideas, crosscutting concepts, and science and engineering practices.^2^ Although originally developed for K-12 science education, many researchers have noted that this framework is also applicable to undergraduate science education, including chemistry.^3,4^ One of the dimensions of three-dimensional learning, scientific and engineering practices, has begun to receive increased attention in the context of undergraduate chemistry education.^5–10^ Though *the NRC Framework* combines science and engineering practices, in this paper, we refer to them as *science practices*, since chemistry is typically regarded as a scientific discipline. Nevertheless, we acknowledge that aspects of engineering practices do manifest at times, especially in the context of chemistry laboratory settings. According to the *NRC Framework*, science is best conceptualized as a collective and iterative process that draws on the collaboration of individuals and institutions. This process includes generating and refining theories, creating models to explain and predict natural phenomena, designing and using instruments for investigation, empirically testing hypotheses, and engaging in discipline-specific forms of communication. It further emphasizes that students should engage in these practices to develop the ability to predict outcomes, construct evidence-based arguments, and model scientific phenomena.

Previous research has demonstrated that engaging in science practices not only improves those competencies but also fosters a deeper epistemological understanding of the practices themselves.^11^ Moreover, engaging in science practices has been shown to increase undergraduate students’ STEM motivation, identity, and sense of achievement.^12^ When students participated in laboratory sessions designed around the practice of argumentation, known as argument-driven inquiry, it was found that they exhibited greater disciplinary engagement and produced more robust arguments.^13^ For chemistry undergraduates, laboratory sessions can serve as valuable spaces that provide ample opportunities to engage in science practices. The American Chemical Society (ACS)^14^ and Royal Society of Chemistry (RSC)^15^ mandate at least 350 h (ACS) or 300 h and a major capstone project (RSC) of laboratory work in the curriculum of a chemistry bachelor’s degree. The 2023 Revised ACS Guidelines have increased emphasis on science practices, specifically the ones developed in laboratory courses.^14^ In short, a chemistry laboratory should be a place where students learn *how to do chemistry*.^16^

Despite the growing emphasis on scientific practices, several reports highlight that the competencies acquired by STEM graduates, including those in chemistry, during their college education are often insufficient to meet workplace demands.^17–21^ A significant, mandated time in the laboratory does not guarantee students will meaningfully engage with science practices - largely due to two factors: (1) the prevalence of “cookbook” experiments and (2) the lack of emphasis in laboratory assessments on evaluating proficiency in scientific practices.^22,23^ In cookbook experiments, students follow a step-by-step procedure to verify a predetermined outcome. While these activities help students develop certain skills, such as using laboratory instruments and collecting data, they do not engage them in essential scientific practices like planning investigations, developing models, and asking questions.^24,25^ Since these structured experiments do not reflect the way scientific investigations are typically conducted, it is crucial to create opportunities for students to develop a broader range of skills in laboratory instruction. Second, laboratory assessments often evaluate the ‘correctness’ of the results obtained rather than students’ proficiency in scientific practices.^26^ However, assessment often dictates students’ learning priorities.^27,28^ If the goal is to develop a proficiency in scientific practices, assessments should explicitly measure these competencies. Previous research underscores the importance of aligning assessments with intended learning outcomes, as misalignment can hinder students from achieving the desired results.^28–33^

This perspective offers the authors’ take on the second aspect—assessment—by discussing limitations of current assessment tools and proposing potential ways to address them.

## Frameworks to Understand Science Practices

Before examining the assessment of scientific practices, it is helpful to first explore ways to conceptualize and categorize them, as this helps to clarify exactly what is being assessed. This step is particularly important given that the term ‘scientific practices’ is not always straightforward and sometimes conflated with the similarly sounding but distinct concept of the scientific method.

Researchers from a broad range of fields have explored science to understand the nature of activities performed by scientists and how they contribute to the creation of scientific knowledge. Kuhn showed that science is conducted by a community of scientists whose work is guided by a set of values and norms, many of which are socially negotiated.^34^ In other words, it is a community of practitioners who follow specific practices that are widely agreed upon and accepted within the scientific field. Since then, it is argued that science should be viewed as a collection of practices, encompassing a range of activities that include specialized forms of communication,^35^ modeling,^36^ and the development of representations of phenomena.^37^ Additionally, these practices are not solely driven by individual cognitive processes; rather, they are shaped and supported by the social, cultural, and material environments in which they occur. Consequently, the environment in which students engage in these practices will itself influence their thinking about science. Therefore, assessment of these practices should consider the actions and interactions in that environment.^38^ *The NRC Framework* outlines a list of eight practices that students must develop to achieve proficiency in science and engineering (listed below).^2^

1. Asking questions and defining problems
2. Developing and using models
3. Planning and carrying out investigations
4. Analyzing and interpreting data
5. Using mathematics and computational thinking
6. Constructing explanations and designing solutions
7. Engaging in argument from evidence
8. Obtaining, evaluating, and communicating information

The framework asserts that acquiring proficiency in these practices helps students become more critical consumers of scientific information. However, listing these practices is just one way to represent the nature of the scientific activities. An alternative approach is to conceptualize them within three broad categories of practices, as illustrated in Figure 1.^2,39,40^

1. Investigating – Scientists ask questions, design experiments, and collect data.
2. Developing Explanations and Solutions – Scientists create models and construct explanations.
3. Evaluating – Scientists critique, compare, and refine ideas through argumentation and peer review.

**Figure 1:**
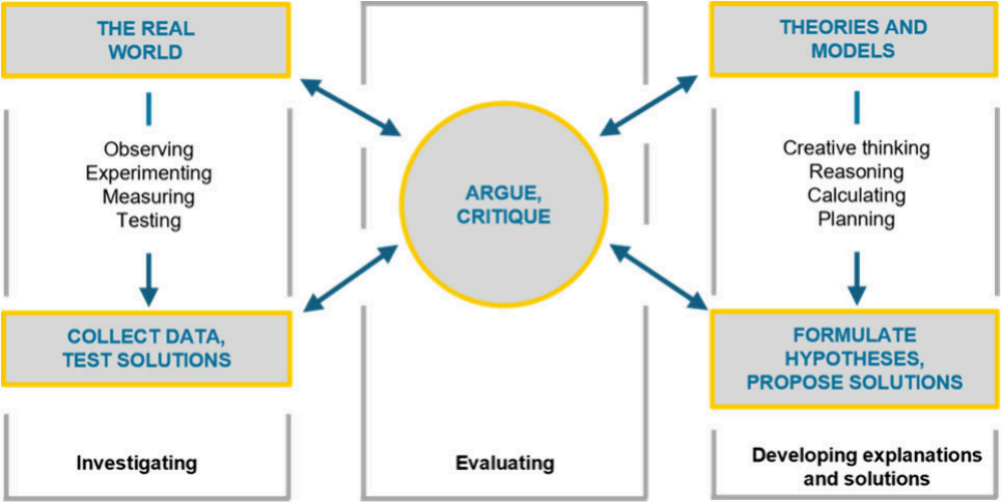
Three broad categories of science practices: Investigating, Evaluating, Developing explanations and solutions^2,39,40^ (Image used with permission).

Different researchers have used various terms to describe these categories of practices, such as investigating, developing explanations and solutions, and evaluating;^39,40^ experimentation, hypothesizing, and evidence evaluation;^41^ investigating, sensemaking, and evaluating and communicating;^42^ investigating, sensemaking, and critiquing.^43–45^ In this work, we will use the terms investigating, sensemaking, and evaluating and communicating (*the ISEC framework*) as we find them to most effectively encapsulate the core aspects of the practices involved. *The ISEC framework* provides a structure for understanding how scientific knowledge is created. For example, most scientific inquiry typically begins with a question, such as ‘How does a molecule’s electronic state affect its acidity?’ These questions lead scientists to develop hypotheses that must be tested, which involve designing experiments and collecting data. These actions generally align with the practice of the investigation. The results of the investigation can contribute to the creation of models and explanatory accounts of phenomena, fitting within the practice of sensemaking. However, achieving consensus on the validity and reliability of the claims made requires argumentation and peer review, which falls under the practice of evaluating and communicating.^39^ At this juncture, it should be emphasized that the *ISEC framework* provides a broad structure intended to encapsulate the practices of science rather than establish a rigid, step-by-step sequence of activities. It should not be misconstrued as a representation of a fixed scientific method. Although the eight practices outlined in the *NRC Framework* and those in the *ISEC framework* may appear different, they are complementary. Several researchers have demonstrated a correspondence between these two frameworks, as illustrated in Figure 2.^42–45^ The investigation phase primarily includes practices of ‘asking questions’, ‘planning and carrying out investigations’, and ‘using computational and mathematical thinking’. The sensemaking phase includes practices such as ‘developing and using models’, ‘analyzing and interpreting data’, and ‘constructing explanations’. Lastly, the evaluating and communicating phase is comprised of ‘engaging in argument from evidence’ and ‘obtaining, evaluating and communicating information’. Based on this correspondence between the two frameworks, we will draw on terms from both depending on the level of granularity under consideration. For instance, we may use the broader term *sensemaking* when addressing competencies such as modeling, data analysis, and constructing explanations. In contrast, if our focus is specifically on data analysis, we will use the term *analyzing and interpreting data*.

**Figure 2:**
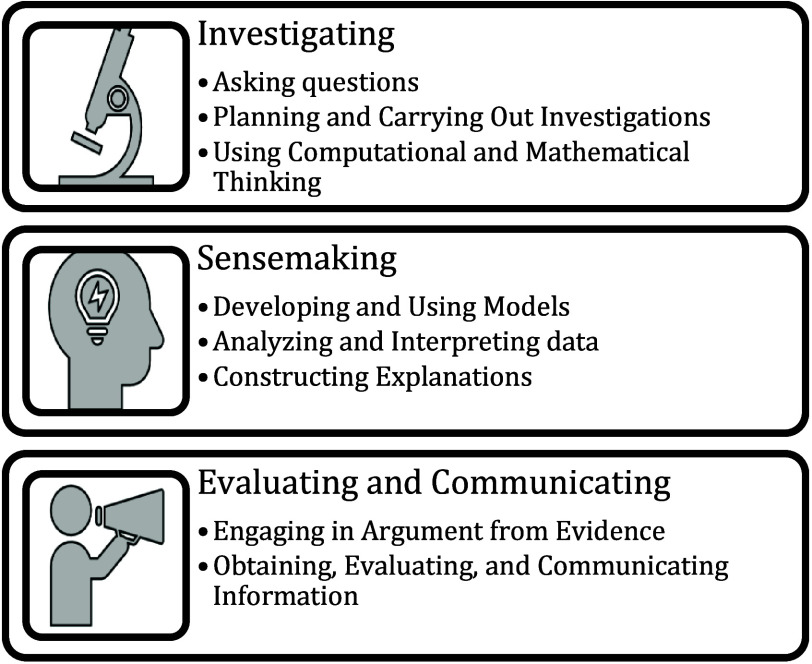
Mapping between the science practices outlined in *the NRC Framework* and *the ISEC framework*.

The categorization of scientific practices across different frameworks can play a key role in informing assessment design, particularly because it is not always feasible to engage students with all of the practices in every laboratory session. Since the focus of this Perspective is on the assessment of scientific practices, we have limited our discussion of the framing of science practices to a select few that are especially relevant. However, scientific practices can be conceptualized in other ways as well, and for a more comprehensive overview of various frameworks, we direct readers to a recent article by Wright et al.^46^

## Assessing Science Practices in Chemistry Labs: Why Do We Need to Do Better?

### Assessing Opportunities to Engage with Science Practices Is Necessary but Not Sufficient

If students are expected to develop proficiency in scientific practices, they must also have opportunities to actively engage with them. Without such opportunities, it is unrealistic to expect the development of these essential competencies. Several studies have analyzed various aspects of the curriculum to evaluate the extent to which students are provided opportunities to engage in scientific practices. One such initiative led to the development of the Three-Dimensional Learning Assessment Protocol (3D-LAP), designed to assess the extent to which evaluation questions promote engagement with the three dimensions of three-dimensional learning.^47^ The science practices component of this protocol was later adapted by Carmel et al. to compare a traditional laboratory curriculum with an inquiry-based approach, demonstrating that the latter offers greater opportunities for student engagement in scientific practices.^7^ Building on this work, Van Wyk et al. further refined the protocol to characterize laboratory experiments in analytical chemistry courses based on their level of inquiry and the extent to which they facilitate engagement in scientific practices.^48^ While these protocols provide valuable insights for instructors in designing experiments and assessments that enhance student participation in scientific practices, they do not directly assess student work or offer feedback for improvement.

### Typical Pre- and In-Lab Assessments Support Skills but Fall Short of Assessing Holistic Engagement

There has been substantial consideration of the assessment of laboratory work, with much of the literature focused on the mismatch between an oft-cited purpose of laboratory work (the development of experimental competencies)^49^ and assessment of laboratory work by report. A major area of focus in shifting this overreliance on reports is the assessment of practical skills directly in the laboratory. Direct assessment of work in the laboratory has been reported in the form of in-laboratory examination of competencies,^50^ postlaboratory instructor assessment of student submitted videos (and awarding of a digital badge for competency) of laboratory techniques,^51,52^ and in-laboratory peer review and postlaboratory instructor review of video recorded laboratory techniques.^53,54^ These approaches typically use rubrics for instructor assessment, with feedback often limited to the extent of achievement of rubric components (with the option of additional comments to highlight specific development points) with the exception of the peer-review approach, which includes additional in-laboratory review by another student. While this approach has been repeatedly demonstrated to be an effective way to structure the assessment of experimental technique competencies in laboratory settings, their developmental goals have meant that their implementation has tended to be a separate component of laboratory work, rather than being integrated into the overall practices of doing an experiment. Moreover, because the focus in these instances is on carrying out specific tasks, such as preparing standard solutions for an experiment, they do not necessarily indicate an understanding of how those tasks relate to the broader purpose or application of the experiment.

### Tools to Assess Engagement with Science Practices Exist but Are Constrained in Scope and Context

A second area of focus in the prior literature is the development of assessment tools to evaluate student engagement with scientific practices in undergraduate chemistry laboratories. Hosbein et al. introduced the IDEAA-GC1 assessment tool, designed for use with the Argument-Driven Inquiry (ADI) curriculum, which focuses on two key scientific practices: engaging in argument from evidence and planning and carrying out investigations.^6^ Similarly, Stephenson et al. developed assessment tasks to evaluate four scientific practices: constructing explanations, engaging in argument from evidence, analyzing and interpreting data, and planning and carrying out investigations in the general chemistry laboratory.^8^ Although these tools are designed to assess scientific practices, they rely on specific tasks developed exclusively for general chemistry laboratories. This limits their applicability in laboratory settings beyond general chemistry. Even within general chemistry, their effectiveness is constrained when laboratory tasks deviate from the prescribed ones. Additionally, these tools assess only a subset of scientific practices, rather than providing a comprehensive evaluation of all practices.

Some tools have been developed to assess the eight scientific practices outlined in *the NRC Framework*.^42,43,55^ Chen et al. introduced the ICAP to Measure by Observation NGSS Science Practice Implementation in the Classroom (IONIC), an observation-based protocol grounded in the Interactive-Constructive-Active-Passive (ICAP) theoretical framework.^42^ The Science Practices Continuum tool was developed using simplified descriptions of each practice to evaluate whole-class engagement in scientific practices.^43^ Additionally, The Systematic Characterization of Inquiry Instruction in Early LearNing Classroom Environments (SCIIENCE) instrument was designed to assess the quality of science instruction in PK-3 classrooms by capturing teacher behaviors and instructional strategies that promote student engagement in science.^55^ While these instruments address all eight scientific practices, their applicability in undergraduate chemistry laboratories is limited. ICONIC is an observation-based protocol designed to assess student engagement by observing students’ behavior in science classrooms. However, practices such as constructing explanations and analyzing and interpreting data at the undergraduate level are often best demonstrated through students’ written artifacts, such as laboratory notebooks and reports. Similarly, the Science Practices Continuum is an observation-based tool developed to assess whole-class engagement, making it unsuitable for evaluating individual students’ engagement. These limitations reduce the applicability of both tools in chemistry laboratory settings, where not all scientific practices are adequately captured through observable behaviors alone. Lastly, the SCIIENCE instrument is designed to assess the quality of science instruction rather than student performance, making it unsuitable for student assessment.

### Feedback Opportunities Exist but Remain Limited and Summative

A third area of consideration is the opportunity for feedback for learning on science practices in the laboratory. It is somewhat surprising, given the volume of literature on laboratory work, the comparative paucity of literature relating to feedback in this domain. Echoing the findings on student intentions and experiences in typical laboratory contexts,^32,33^ the exploration of student experience on the utility and value of feedback in these contexts finds that there is a tendency to focus on summative (that is to say grades) rather than formative (developmental) aspects of feedback.^56^ Efforts to engage students in feedback have included peer feedback activities,^57^ the use of evaluative judgements in a research project,^58^ and the use of rubrics to promote self-assessment.^59^ However, the scant literature in the area of feedback generally and in relation to science practices, more specifically in the context of general laboratory teaching approaches, highlights the need for more consideration in this domain.

## Assessing Science Practices in Chemistry Labs: How Can We Do Better?

As discussed above, assessment in the laboratory can take various forms, including written documents, quizzes, practical exams, and peer evaluations. A common approach involves evaluating students’ performance through direct observation and by reviewing their laboratory notebooks and reports. However, for an assessment tool designed for laboratory use to be effective in facilitating and documenting growth, it must incorporate several key features and be applicable to a range of contexts. An overview of innovative laboratory approaches from the past decade highlighted the breadth of considerations and contexts laboratory work encompasses and in which scientific practices are situated. This overview, parsed as “10 guiding principles”, highlighted several factors such as the need to situate work in particular contexts, allowing for independent structured inquiry, consideration of safety and sustainability, as well as provision of opportunity for students to reflect on and document their own learning.^60^ Assessment of science practices needs to be considered in a way that is robust enough to be modified for this variety of contexts. Below, we outline important considerations for developing assessments specifically for use in this broad range of undergraduate chemistry laboratory settings.

### Assessments Should Explicitly Measure the Competencies We Intend Students to Achieve

A key consideration when developing assessments should be their alignment with learning outcomes, an idea rooted in the principle of constructive alignment. This model consists of three key components: (1) intended learning outcomes, (2) student tasks, and (3) assessments.^28,29^ The core premise is that when student tasks and assessments are aligned with the intended learning outcomes, students are more likely to achieve those outcomes. For instance, if an instructor aims to cultivate students’ proficiency in scientific investigation, then learning activities should provide authentic opportunities to practice investigation, while assessments should evaluate students’ investigative skills and offer constructive feedback to support their growth. A few studies in the past have focused on structuring learning activities such that they increase student engagement with science practices. Work in this domain includes the redesign of prelaboratory questions to situate them explicitly in science practices relating to planning and carrying out investigations,^61^ the building in of dialogue prompts relating to planning inquiry activities,^62^ or the use of preparatory work to inform subsequent decision-making in the laboratory.^63^ These approaches and many others like them introduce novel and valuable ways to incorporate the inclusion of science practices into the laboratory curriculum, but as told, they do not elaborate on the extent to which those science practices are assessed in a substantive way and consequently are not clear on the extent to which students can use feedback from this work to guide their ongoing development in science practices. Therefore, it is necessary to align not only the learning activities but also assessments with learning objectives.

As a specific example, consider an instructor who wants students to develop the ability to plan an investigation. Learning activities in this case should allow students to select appropriate methods such as instruments and reagents, account for safety considerations and risk-minimization strategies, and identify suitable methods for data collection and analysis. Correspondingly, assessments should measure how effectively students planned their investigations, focusing on scientific practice competencies such as the feasibility of selected methods, the adequacy of safety considerations, and the justification of methodological choices. Too often, assessments emphasize only the results of an investigation, overlooking the essential process skills that enable students to engage in scientific practices. Furthermore, because assessments heavily influence what students prioritize in their learning, ensuring alignment between tasks, assessments, and learning objectives is critical for promoting meaningful skill development.^27,28^

Of the many different formats assessments can take, rubrics are often used as scoring guides to assign grades. These rubrics should be designed to ensure alignment between the intended outcomes and assessments. The highest performance level in each rubric category can be aligned with key learning outcomes that students are expected to achieve.^64^ By incorporating generalized performance descriptors across various levels of competencies, rubrics can serve as essential resources for both instructors and students, facilitating structured assessment, self-evaluation, and meaningful feedback for continuous development while being adaptable to specific contexts. Researchers from the Enhancing Learning by Improving Process Skills in STEM (ELIPSS) project have developed rubrics to assess essential process skills for undergraduate STEM students, including critical thinking and information processing.^59,65^ These rubrics could serve as useful templates for developing similar tools focused on assessing scientific practices.

However, even when assessments are thoughtfully designed to focus on skills, it can be challenging to ensure that students provide evidence of the practices. This requires careful consideration of the task design and often explicit prompting for students to record their process. Additionally, given the less well-defined nature of assessing skills, in contrast to checking whether a “correct answer” was achieved, there can be significant variation in instructor grading, particularly in large courses where much of the work is done by graduate teaching assistants.^66^ This may require additional effort to calibrate the evaluators.

### Assessments Should Support Instructors in Their Design of Instructional Approach

While the primary purpose of assessments is to assess student work and provide feedback, they have the potential to serve as a valuable tool for instructors to evaluate the effectiveness of their teaching strategies. By clearly defining expectations and performance criteria, assessments help inform instructional strategies and curricular improvements.^67^ Analyzing assessment results allows instructors to gain insights into student learning, helping them refine their teaching approaches to better meet student needs. In the context of science practices, assessments can help instructors identify areas where students need additional support and design meaningful opportunities for active engagement. For example, if assessments of laboratory reports reveal that students have not adequately supported their claims in the discussion section, instructors can address this by teaching strategies for constructing arguments before the laboratory or by providing scaffolds in the laboratory manual, such as dividing the discussion section into claim, evidence, and reasoning. These supports can help students develop stronger evidence-based arguments. Furthermore, in laboratory settings, assessments have traditionally focused on investigation-related practices, such as describing experimental procedures, making and recording observations, and collecting data,^68^ while practices related to sensemaking, evaluation, and communication, such as critiquing peers’ arguments or developing models to explain experimental results, have received comparatively less attention.^39,44,69^ However, we recognize that even when assessments indicate areas to be addressed, institutional and departmental factors also play a significant role in curricular changes. When courses are taught by multiple instructors, a consensus must be achieved to make sustained curricular changes. For courses with more complex structures, such as the coordination of undergraduate or graduate teaching assistants, changes may also require additional training.

### Assessments Should Be Applicable and Adaptable to a Variety of Undergraduate Chemistry Laboratory Experiences

Scientific practices are essential competencies that must be assessed across a range of learning environments—including laboratories, classrooms, and fieldwork. With careful design considerations, it is possible to develop assessments that are well-suited for use in laboratory settings. Moreover, since the laboratory is an environment where many scientific practices are expected to manifest, assessments tailored for this context can serve as valuable resources for the teaching community.

One of the key limitations of existing assessment tools for science practices is their narrow scope, often tailored to a single type of laboratory course, such as general chemistry, and for a narrowly defined task.^6,8^ To address this, there is a need for assessments that can be broadly applicable across undergraduate chemistry laboratories, as the core nature of scientific practices remains consistent, even as their complexity increases with student experience^70^ and academic progression. For example, in the practice of argumentation, students are expected to make claims, present evidence, and construct logically coherent arguments, whether they are freshmen in an introductory laboratory or seniors in an advanced organic chemistry course. What distinguishes these contexts is not the fundamental practice itself but rather the level of complexity and the nature and depth of the chemistry content knowledge: more advanced students may be expected to integrate multiple forms of evidence and demonstrate nuanced reasoning within the specific chemistry content they are studying, whereas beginning students may be required to support their claims with only a single piece of evidence tied to their course content. Despite these differences, the underlying structure of the practice—making claims, presenting evidence, and constructing arguments—remains stable. Consequently, assessments can be designed to retain stable descriptors that capture the essence of the practice, while the rating scale or performance criteria can be adapted to reflect differences in the expected complexity and content knowledge across course levels. When used this way, a freshman who constructs a clear, basic argument may receive a high score, while a senior presenting an equally simple argument, when greater sophistication is expected, may receive a lower score by using the same assessment tool.

While we have emphasized the importance of assessing engagement with scientific practice in laboratory settings, it may not be realistic for instructors to focus on all practices in every laboratory session. The emphasis on particular practices will naturally vary depending on the nature of the laboratory course. Some laboratory courses may lend themselves more readily to the development of specific practices, while others may support different aspects of scientific engagement. For example, analytical or physical chemistry laboratories may offer more opportunities to foster mathematical and computational thinking, whereas an organic chemistry laboratory may better support the development of argumentation skills, particularly when students are required to justify the structure of a compound synthesized by using multiple sources of evidence, such as NMR, IR, and mass spectrometry. Therefore, for assessments to be truly effective, their design must be flexible and customizable, allowing instructors to adapt them to suit the specific needs of their courses.

Lastly, while science practices are generally consistent across a variety of laboratory environments, certain aspects are uniquely emphasized within the discipline of chemistry, for example, chemical safety. Safety considerations, such as hazard identification, risk assessment, and the implementation of appropriate mitigation strategies, are central to the practice of chemistry and therefore should be incorporated during the planning phase of the experiment. Consequently, assessments designed for science practices in undergraduate chemistry laboratories must explicitly address these discipline-specific competencies. Ensuring some consistency across different chemistry laboratory courses to support skill development throughout the program of study requires communication and coordination among multiple course instructors. This type of structure is not necessarily the norm in many departments.

### Assessments Should Be Able to Assess Written Artifacts and Student Behaviors

As previously mentioned, scientific practices often manifest in ways that require the assessment of both student behaviors and written artifacts. Certain aspects of student engagement, particularly those related to laboratory safety protocols or the proper use of instrumentation, require direct observation to determine whether students are performing their tasks in a manner consistent with established standards. In contrast, many other scientific practices—such as using models, analyzing and interpreting data, and constructing evidence-based arguments—are primarily demonstrated through written work such as laboratory reports. However, in many laboratory courses, the assessment of student work tends to prioritize the accuracy of results over the skills and processes involved in achieving them. Conversely, student behaviors are often overlooked in assessment^16,71^ or assessed only as a part of summative evaluation, such as laboratory practicals, limiting opportunities for feedback and improvement.^50,72^ The current tools to assess science practices are typically designed to evaluate either written work, such as IDEAA-GC,^6^ or student behavior through observation, like ICONIC.^42^ To comprehensively assess scientific practices, assessments must be designed to evaluate both written and observed elements of student performance depending on the specific practice under consideration. However, assessing competencies during experiments presents challenges such as dividing attention between instruction and evaluation and the potential for students to feel they are being constantly observed. Given that laboratory sessions typically last 2–3 h, assessments can be scheduled to occupy only a portion of this time and conducted during noninstructional phases, helping to mitigate some of these concerns. Assessing behavior in the moment rather than in a document also requires a means of quickly recording the observed level of performance. Simpler methods of recording assessments, such as using a digital tablet, will be needed to further streamline the process of documenting student behaviors. This is currently an underexplored area in laboratory education research.

### Assessment Should Provide Targeted Feedback for Reflection and Improvement

Assessment without feedback is merely an evaluation. Regardless of the form it takes, assessment should provide students with clear criteria for success and opportunities for reflection, enabling them to improve when they engage in similar tasks in the future. This idea is rooted in the principle of self-regulated learning (SRL), which posits that learning occurs through a cyclical process comprising three key phases: (1) forethought, (2) performance, and (3) reflection.^73^ In the *forethought* phase, students plan their tasks; during *performance*, they actively engage in those tasks while considering the purpose behind each step; and in the *reflection* phase, they evaluate their performance to identify areas for improvement. This iterative cycle continues as students apply insights gained from previous experiences to their future learning. However, students often need assistance during the reflection phase to accurately identify areas of growth and how to improve. This is where feedback as part of the assessment becomes essential. Instructors and teaching assistants play a crucial role in this process by providing feedback that guides students through the learning cycle.^74^ Schunk and Swartz have demonstrated that students achieve significant learning gains when they receive feedback on their learning process.^75^ The structure of the course should also support student engagement with feedback; otherwise, students tend to focus on the score rather than opportunities for growth and improvement.^56^ This highlights the importance of providing targeted feedback to support the development of proficiency in scientific practices.

When developing assessments, it is important to embed targeted suggestions that instructors can offer to students to help them strengthen the specific practice under consideration. As was noted by Jørgensen et al.,^56^ the scoring and feedback need to have sufficient detail about the quality of the student’s work and provide guidance for how the students can improve their work, so that students can find the feedback useful. During the assessment process, depending on the level of proficiency demonstrated, instructors can direct students toward relevant suggestions that address areas of weakness. As previously noted, researchers from the ELIPSS project have developed rubrics for assessing process skills such as critical thinking and information processing.^59,65^ A distinguishing feature of these rubrics is the inclusion of a section dedicated to suggestions for improvement, which helps students focus on specific aspects requiring further development. Used in this way, assessments become powerful tools for feedback, supporting students during the reflection phase of the self-regulated learning cycle. For example, if the assessment tool to evaluate students’ safety planning includes resources or descriptions on recognizing hazards and minimizing risks, instructors can use them to guide students to the areas where they need improvement. However, because it is impossible to anticipate every way a student may require feedback, these embedded suggestions can address only some needs, and individualized feedback may still be necessary.

## Conclusion

“If you ask students to make a hypothesis but do not assess it, then they have no reason to learn to make a hypothesis.” This sentiment, expressed by a graduate chemistry laboratory assistant, resonates with many stakeholders involved in laboratory instruction. Increasingly, questions have been raised—rightfully so—about the effectiveness of laboratory instruction, especially considering the significant time investment and the high costs associated with infrastructure and consumables.^71,76^ If we are to justify the inclusion of laboratory work within the chemistry curriculum, the laboratory must be a space where students learn how to *do* chemistry.^16^ Learning to do chemistry involves more than following procedures from a ‘cookbook’ experiment or mastering the use of instruments; it must be a space primarily dedicated to the development of essential competencies. Achieving this goal requires providing meaningful opportunities for students to engage with scientific practices and explicitly assessing them.

However, even when instructors wish to assess these competencies, there is a shortage of assessment tools that can be broadly applied across different undergraduate chemistry laboratories. Moreover, existing tools often fail to comprehensively assess the full range of scientific practices or provide actionable feedback for student improvement. In this paper, we outline five recommendations for developing effective assessment tools that promote skill development in the laboratory, which are summarized in Table 1.

**Table 1:** Compilation of Recommendations Relating to Assessments of Science Practices

Recommendation	Consideration for practice
1. Assessments should explicitly measure the competencies we intend students to achieve.	•Align learning goals, student tasks, and assessments to meaningfully integrate science practices into laboratory work.
	•Emphasize assessment of aspects of process relating to practices rather than just the product.
	•Use intentionally designed rubrics to assess process skills.^59,65^
2. Assessments should support instructors in their design of instructional approach.	•Use assessment as a tool for the evaluation of teaching strategies.
	•Use a more granular focus on practices to identify areas where students need support.
	•Use the ISEC Framework to identify more general practices relating to (i) investigating, (ii) sensemaking, and (iii) evaluating and communicating where students may need more scaffolding and support.^77^
3. Assessments should be applicable and adaptable to a variety of undergraduate chemistry laboratory experiences.	•Track student progress through a curriculum to increase student engagement in similar practices in a variety of increasingly complex contexts.
	•Identify the core practices for the purposes of assessment to help students identify and consolidate these practices, even as the context changes.
	•Draw on examples eliciting core practices in particular contexts^6,8^ and subsequently determine the extent to which the level of sophistication of evidencing that practice may evolve in different contexts.
4. Assessments should be able to assess written artifacts and student behaviors.	•With a focus on the processes involved in science practices, determine when both written artifacts and student behaviors provide evidence for practices.
	•Assess behaviors such as use of instrumentation and laboratory safety in ways that do not overburden students with the sense of being continually observed.^50,72^
5. Assessment should provide targeted feedback for reflection and improvement.	•Feedback is an essential component of the learning process; include prompts that enable students to evaluate their performance in preparation for future learning.
	•Provide specific detail on science practices under consideration to improve the helpfulness of feedback from a student perspective.^56^
	•Use rubrics that include particular prompts or directions on targeted areas as a means to embed the future support in the assessment infrastructure.^59,65^

While it may be desirable to incorporate all recommendations when designing assessments, we argue that three core elements are essential: explicit measurement of competencies, evaluation of artifacts and behaviors, and feedback that supports reflection and improvement. Depending on the nature and goals of a given laboratory course, other aspects may be emphasized to varying degrees. We also propose that rubrics as assessment tools offer a viable and practical approach to achieving these intended outcomes, whether used during experimentation or applied to written work, due to their efficiency, ability to measure specific competencies, and capacity to deliver constructive feedback.^78,79^ By ensuring that learning outcomes, assessments, and instructional actions are aligned, instructors provide students with coherent curricular opportunities to develop their knowledge and skills and increase the likelihood that instructional actions are appropriate for achieving the desired learning outcomes. Constructive alignment that supports achievement of science practices in chemistry courses requires appropriate tasks designed to engage students in science practices and approaches to assessment that provide information to both students and instructors on the practice itself.
